# Cantharidin increases the force of contraction and protein phosphorylation in isolated human atria

**DOI:** 10.1007/s00210-023-02483-9

**Published:** 2023-04-25

**Authors:** R. Schwarz, B. Hofmann, U. Gergs, J. Neumann

**Affiliations:** 1 Institute for Pharmacology and Toxicology, Medical Faculty, Martin Luther University Halle-Wittenberg, Magdeburger Straße 4, 06112 Halle (Saale), Germany; 2 Department of Cardiac Surgery, Mid-German Heart Center, University Hospital Halle, Halle (Saale), Germany

**Keywords:** Cantharidin, Human atrium, Mouse atrium, Phosphatases, Phosphorylation

## Abstract

Cantharidin, an inhibitor of protein phosphatase 1 (PP1) and protein phosphatase 2A (PP2A), is known to increase the force of contraction and shorten the time to relaxation in human ventricular preparations. We hypothesized that cantharidin has similar positive inotropic effects in human right atrial appendage (RAA) preparations. RAA were obtained during bypass surgery performed on human patients. These trabeculae were mounted in organ baths and electrically stimulated at 1 Hz. For comparison, we studied isolated electrically stimulated left atrial (LA) preparations and isolated spontaneously beating right atrial (RA) preparations from wild-type mice. Cumulatively applied (starting at 10 to 30 µM), cantharidin exerted a positive concentration-dependent inotropic effect that plateaued at 300 µM in the RAA, LA, and RA preparations. This positive inotropic effect was accompanied by a shortening of the time to relaxation in human atrial preparations (HAPs). Notably, cantharidin did not alter the beating rate in the RA preparations. Furthermore, cantharidin (100 µM) increased the phosphorylation state of phospholamban and the inhibitory subunit of troponin I in RAA preparations, which may account for the faster relaxation observed. The generated data indicate that PP1 and/or PP2A play a functional role in human atrial contractility.

## Introduction

**Fig. 1:**
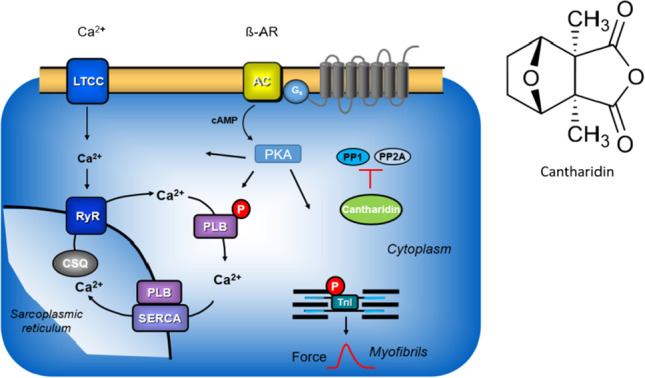
Mechanism(s) of action of cantharidin in cardiomyocytes. β-adrenergic stimulation augments the activity of adenylyl cyclases (AC) in the sarcolemma via stimulatory guanosine-triphosphate (GTP)-binding proteins (Gs) and more formation of 3´, 5´ -cyclic adenosine monophosphate (cAMP). Thereafter, cAMP activates protein kinases that phosphorylate (PKA) and thereby activate regulatory proteins. β-adrenergic stimulation increases cytosolic Ca^2+^ that activates the myofibrils. Using a different mechanism namely enhanced activity of a MLC-kinase, the phosphorylation of myosin light chain 20 (MLC20) increases the affinity of the myofilaments for Ca^2+^and thereby enhances force of contraction. ^l^Ca^2+^ can pass through the L-type Ca^2+^ -channel (LTCC). Ca^2+^ can be released from the sarcoplasmic reticulum (SR) via ryanodine receptors (RYR). Phosphorylation of phospholamban (PLB) de-inhibits the activity of the SR—Ca^2+−^ATPase (SERCA) thus facilitating relaxation. Phosphorylation the inhibitory subunit troponin (TnI) reduces the Ca.^2+^-sensitivity of myofilaments also hastening relaxation. PLB, MLC20 and TnI can all be dephosphorylated by PP1 and PP2A. The enzymatic activities of PP1 and PP2A can inhibited by cantharidin (see structural formula in inset, a naturally occurring molecule)

There is ample evidence of the expression of protein phosphatases in the human heart. For instance, β-adrenergic stimulation increases the force of contraction and the beating rate in the human heart (Fig. 1). Contemporary theories explain the mechanism underpinning these effects based on a signal transduction system involving the activation of β-adrenoceptors, the activation of adenylate cyclase via stimulatory guanosine-triphosphate (GTP) binding proteins, and the formation of 3´ 5´-cyclic adenosine monophosphate (cAMP). Subsequently, in the working myocardium, cAMP activates kinases that phosphorylate—e.g., protein kinase (PKA)—and thereby activates regulatory proteins. The inotropic effects of β-adrenergic stimulation are ultimately explained by an increase in cytosolic Ca^2+^ that activates the cardia myofilaments. These phosphorylations are reversed dynamically; in this way, the inotropic effect of β-adrenergic stimulation is in this way by protein phosphatases (Gombosova et al. 1998; Neumann et al. 1999; Herzig and Neumann 2000). In this study, we focus on PP1 and PP2A because they constitute the bulk of cardiac protein phosphatases and are both able to dephosphorylate phospholamban and troponin I (Herzig and Neumann 2000, Neumann et al. 2021, Wijnker et al. 2011, Gergs et al. 2019b, Neumann et al. 1999). We have demonstrated in previous studies that cantharidin inhibits the purified catalytic subunits of PP1 and PP2A in guinea pig and human cardiac preparations (Neumann et al. 1995a, b; Linck et al. 1996). Furthermore, in guinea pig ventricular cardiomyocytes labelled with radioactive orthophosphate (^32^P), we detected that cantharidin induces a time- and concentration-dependent increase in radioactivity in several cardiac proteins (Neumann et al. 1995a, b), among which we identified phospholamban and troponin I (TnI), the inhibitory subunit of troponin (Neumann et al. 1995a, b). We observed that cantharidin increases the force of contraction in guinea pig papillary muscles and in human ventricular preparations (Neumann et al. 1995a, b; Linck et al. 1996). To the best of our knowledge, there is no published study on the effects of cantharidin in human atrial preparations or mouse atrial preparations—investigated in this study for comparison. Similarly, cantharidin increases the contractility of the cells (thereby enlarging cell wall motion), augments the current flowing through the L-type Ca^2+^ channel and increases the cytosolic Ca^2+^ concentration (Ca^2+^ transient) in guinea pig ventricular cardiomyocytes (Neumann et al. 1995a, b; Bokník et al. 2000). We have shown that cantharidin also increases phospholamban and TnI phosphorylation in isolated perfused rat hearts (Bokník et al. 2001). Other studies have extended our data with interesting findings. For instance, it has been reported that, at 30 µM, cantharidin begins to exert a positive inotropic effect in isolated dog ventricle trabeculae (Chu et al. 2003) and cantharidin increases the positive inotropic effect of noradrenaline in isolated dog ventricle trabeculae (Chu et al. 2003).

Interestingly, in quiescent cardiomyocytes from guinea pigs, isoprenaline increases phospholamban phosphorylation only at amino acid serine 16 in phospholamban, while cantharidin augments the phosphorylation state of phospholamban on amino acid serine 16 and amino acid threonine 17 under the same conditions, indicating constant protein phosphatase activity at these amino acids in phospholamban (Bokník et al. 2001). We observed a good temporal correlation between the relaxant effects of cantharidin and phospholamban serine 16 phosphorylation in guinea pig papillary muscles, suggesting a causal relationship (Bokník et al. 2001).

We tested the following main hypotheses: (1) cantharidin increases the phosphorylation of significant regulatory proteins in the human atrium. (2) Cantharidin increases the force of contraction in the human atrium. (3) Cantharidin increases relaxation in the human atrium. Progress reports on this research have previously been published as abstracts (Schwarz et al. 2022, 2023).

## Materials and methods

### Contractile studies on mice

Right and left atrial preparations from mice were isolated and mounted in organ baths, as described in previous studies (Gergs et al. 2013; Neumann et al. 2003). The bathing solution in the organ baths contained 119.8 mM NaCI, 5.4 mM KCI, 1.8 mM CaCl_2_, 1.05 mM MgCl_2_, 0.42 mM NaH_2_PO_4_, 22.6 mM NaHCO_3_, 0.05 mM Na_2_EDTA, 0.28 mM ascorbic acid and 5.05 mM glucose. The bathing solution was gassed continuously with 95% O_2_ and 5% CO_2_ and maintained at 37 °C and pH 7.4 (Neumann et al. 1998, 2003; Kirchhefer et al. 2004). Spontaneously beating right atrial preparations from mice were used to study chronotropic effects.

Drug application was as follows: after equilibration was achieved, cantharidin was cumulatively added to the left atrial and right atrial preparations to establish concentration–response curves. Then, where indicated, carbachol was cumulatively applied to the preparations. Finally, after the response stabilized and without any washout, in some experiments 1 µM isoprenaline was added to test the efficacy. Moreover, in some studies we established cumulative concentration response curves to isoprenaline (0.1 nM to 1 µM) in the presence and absence of 30 µM cantharidin. In addition, we freeze clamped beating left atrial preparations in the presence of 100 µM cantharidin after the positive inotropic effect of cantharidin had reached a stable plateau.

### Contractile studies on human atrial preparations

Contractile studies were performed on human atrial preparations using the same setup and buffer used in the mouse studies (Sect. 2.1). The samples were obtained from nine male patients and four female patients aged 34–82 years. The drug therapy included metoprolol, furosemide, apixaban and acetyl salicylic acid. The methods we used for the atrial contraction studies conducted on human samples have been described in a previous study and were not altered in this study (Gergs et al. 2009, Gergs et al 2018; Boknik et al. 2019). We froze the samples from the human atrium in liquid nitrogen and then subjected them to Western blotting (Sect. 2.3). Moreover, in some studies we established cumulative concentration response curves to isoprenaline (0.1 nM to 1 µM) in the presence and absence of 100 µM cantharidin.

### Western blotting

Homogenization of the samples, protein measurements, electrophoresis, primary and secondary antibody incubation and quantification were performed as per our previously established protocols (Gergs et al. 2009, 2019a,b; Boknik et al. 2018). We homogenized the frozen samples, separated their proteins using denaturing gel electrophoresis, and then transferred the proteins to membranes via electrophoresis. The membranes were incubated with specific first antibodies, and subsequently, with second antibodies. The first antibodies used were: anti-calsequestrin (CSQ) antibody, Santa Cruz (1:20,000; Cat. # sc390999), anti-phospholamban (pSer16) antibody (1:5000; Badrilla, Leeds, UK, Cat. # A-010–12), anti myosin light chain (MLC) antibody (1:5000; Abam, Cambridge, MA, USA, Cat. # ab2480), and anti phospho-troponin I (P-TnI) antibody, (1:5000; Cell signalling, Danvers, MA, USA, Cat. # 4004).

### Data analysis

The data are presented as the mean ± standard error of the mean. The statistical significance was estimated using an analysis of variance, followed by the Bonferroni *t*-test, and a *p*-value of < 0.05 was considered significant.

### Drugs and materials

The drugs, isoprenaline-hydrochloride, cantharidin (CANT, 100 mM in dissolved dimethylsulphoxide (DMSO)), and carbachol (CAR), were purchased from Sigma-Aldrich (Steinheim, Germany). All other chemicals used in this study were of the highest purity grade commercially available. Deionized water was used throughout the experiments, and the stock solutions were freshly prepared daily.

## Results

**Fig. 2:**
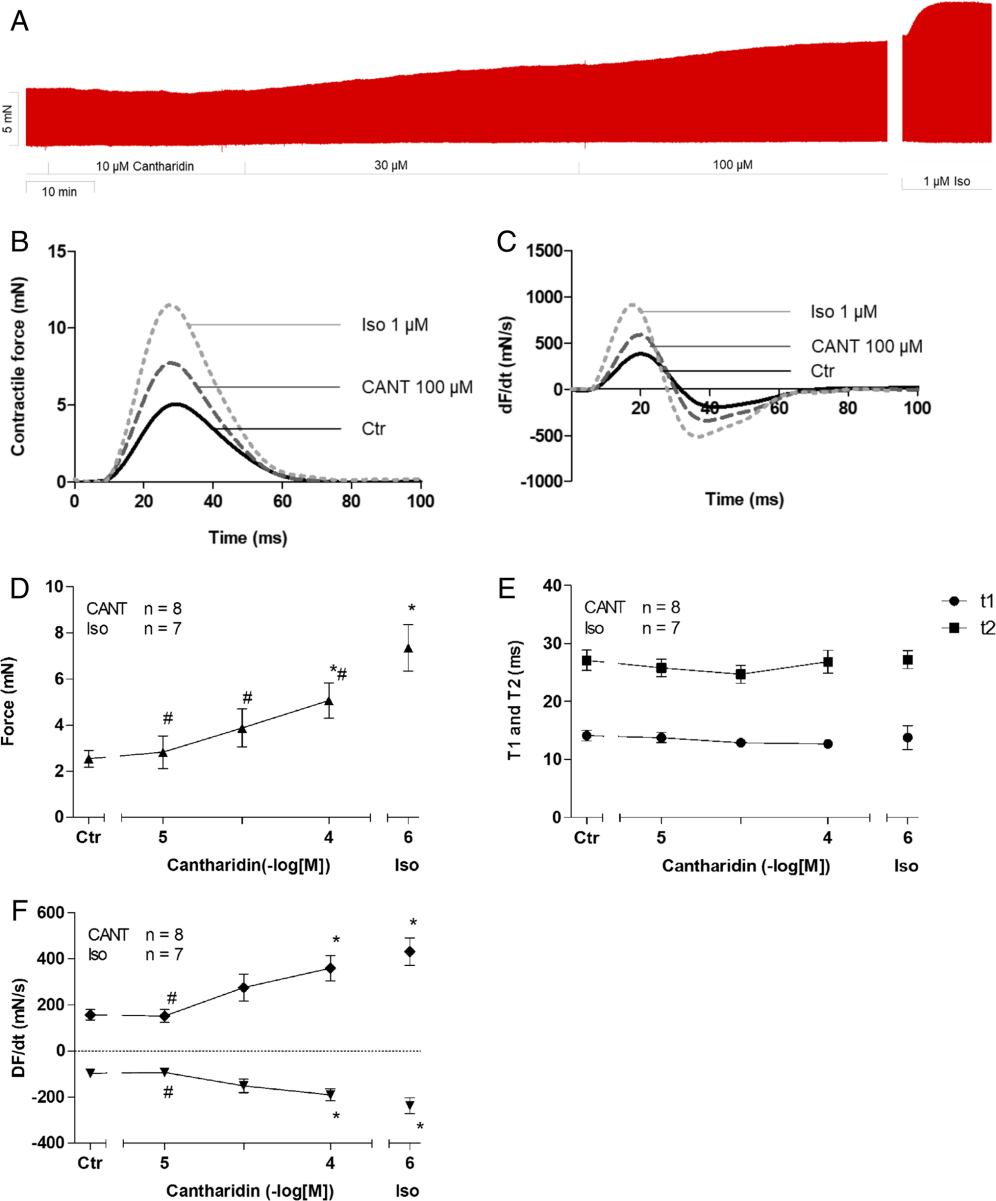
Cantharidin increases force of contraction in mouse left atrium. A: Original recording in mouse left atrial preparations. It becomes apparent that cantharidin induced as time- and concentration-dependent positive inotropic effect. B: original recordings as in A but here superimposed force tracings at high temporal resolution in milli seconds are shown (ms). Here, the increase in force by cantharidin compared to pre-drug values can be evaluated (Ctr). C: original recordings superimposed tracings of the first derivative of force versus time at high temporal resolution in mN/ms. Here, the increase the positive and negative first derivative of force with respect to time (dF/dt) by cantharidin compared to pre-drug values is plotted. Summarized concentration–response curve for the effect of cantharidin on force of contraction (D), time to peak tension (E: T1) and time to relaxation (E: T2), rate of tension development (F: dF/dt_max_) and rate of tension relaxation (F: dF/dt_min_). * *p* < 0.05 vs. CTR, # *p* < 0.05 vs. isoprenaline (Iso). Ordinates in A and B: Force of contraction in milli Newton (mN). Ordinates in A and B in mN. Ordinates in C and D in milli seconds (ms). Rate of contraction and rate of relaxation in E mN/s. Abscissae indicate concentrations of cantharidin in negative decadic concentrations. Horizontal bar in A indicates time axis in minutes (min). Significant differences versus control (CTR; pre-drug value) is indicated in asterisks. “n” indicates number of experiments

Cantharidin increased the force of contraction in isolated electrically paced left atrial preparations from human patients in a time- and concentration-dependent manner (Fig. 2A). The positive inotropic effect of cantharidin became observable at 10 µM (Fig. 2D). We measured the contractile force when it plateaued, which occurred after 15 min. A subsequent higher concentration of cantharidin was then added. The contractile parameters were measured just before the next concentration of cantharidin was added. The potency of cantharidin observed in these isolated electrically paced left atrial preparations from human patients is similar to the potency observed in left atrial preparations from guinea pigs (Neumann et al. 1995a, b). At a high temporal resolution, inotropic effects can be superimposed (Fig. 2B, Fig. 2C). Summarizing these data, we observed an increase in the tension developed (Fig. 2D) at concentrations as high as 100 µM (Fig. 2D). Cantharidin neither shortened nor prolonged the time to relaxation (t2, Fig. 2E) nor the time to peak tension (t1, Fig. 2E). However, cantharidin increased the absolute values of the rate of tension development and the rate of relaxation in the left atrial preparations (Fig. 2F).

**Fig. 3:**
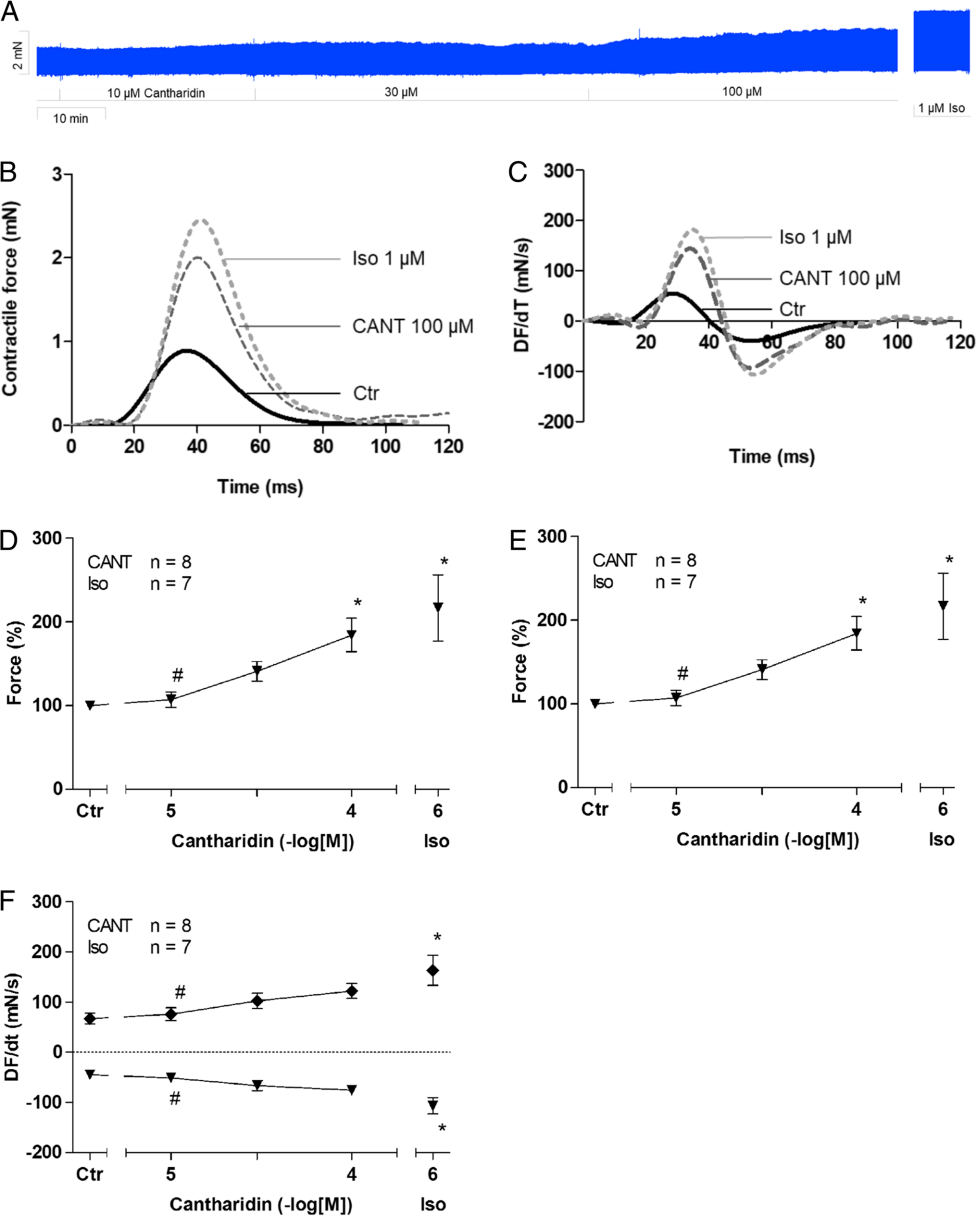
Cantharidin increases force of contraction in mouse right atrium. A: Original recording of developed tension in electrically stimulated right atrial preparation, B: original recordings: superimposed force tracings at high temporal resolution in milli seconds (ms). Here, the increase in force by 100 µM cantharidin is compared to pre-drug values (Ctr).C: original recordings superimposed tracings of the first derivative of force versus time at high temporal resolution in mN/ms. Here, the increase in force by cantharidin was compared to pre-drug values D: Concentration response curve for the effect of cantharidin on force of contraction and time to peak tension (E) and time to relaxation (E): rate of tension development (F: dF/dt_max_) and rate of tension relaxation (F: dF/dt_min_). * *p* < 0.05 vs. Ctr, # *p* < 0.05 vs. isoprenaline (Iso). Ordinate in 3A and 3B: force of contraction in milli Newton (mN). Ordinate in 3A in mN. Ordinate in 3D in % of control (Ctr: no drug addition: 1.40 mN ± 0.45 mN). Ordinate in 3E in milli seconds (ms) and in 3F in mN/ms. Rate of contraction and rate of relaxation in 3C and 3F mN/s. Abscissae indicate concentrations of cantharidin in negative decadic concentrations. Horizontal bar in A indicates time axis in minutes (min). Significant differences versus control (Ctr; pre-drug value) is indicated in asterisks. “n” indicates number of experiments

**Fig. 4:**
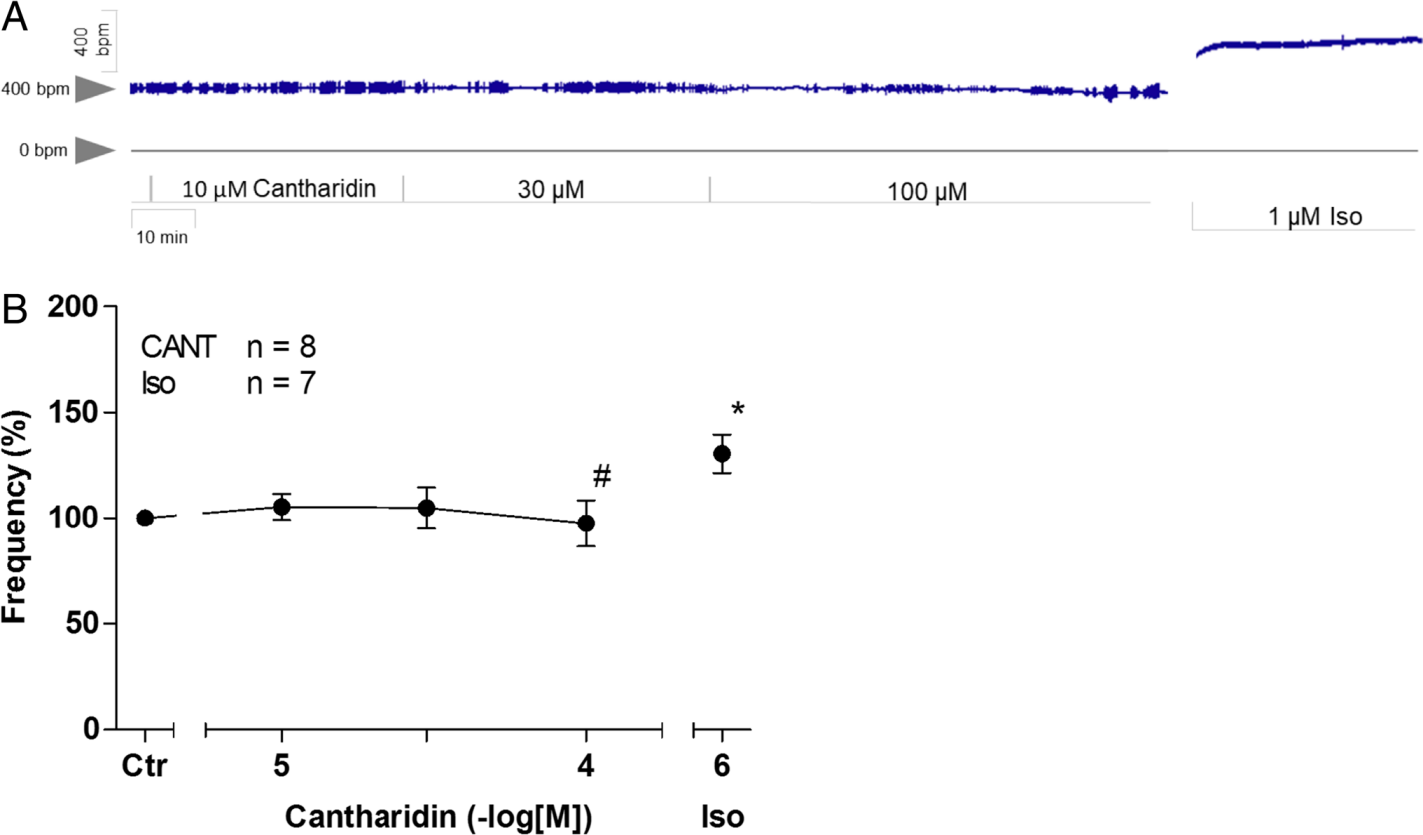
Cantharidin does not increase the beating rate. A: Original recording of the effect of cantharidin (CANT) on beating rate in beats per minute (bpm Ordinate) over time in minutes (min, Abscissa). At the end samples were washed repeatedly and isoprenaline was added. For comparison the effect of isoprenaline is shown. B: Ordinate: summarized values in % of control (Ctr: no drug addition: 392 bpm ± 31.4 bpm). “n” indicates the number of experiments. Ctr: pre-drug value. Abscissa: concentration of cantharidin or corresponding volume of solvent was added. For comparison the effect of isoprenaline is shown. * *p* < 0.05 vs. Ctr, # *p* < 0.05 vs. isoprenaline (Iso)

Similar to observations in paced left atrial preparations, cantharidin also augmented the force of contraction in spontaneously beating right atrial preparations. This can be seen in an original recording at low chart speed (Fig. 3A, Fig. 3B) and at high chart speed (tracings of single contractions, Fig. 3B), proving that cantharidin is active in right atrial preparations. Similar to its effect in left atrial preparations, cantharidin failed to alter time to peak tension or the time to relaxation (Fig. 3E), but cantharidin augmented the rate of tension development and the rate of relaxation, as can be seen in the original recordings presented in Fig. 3C and summarized in Fig. 3F. These observations are similar to those in the data from our previous study on isolated spontaneously beating right atrial preparations from guinea pigs (Neumann et al. 1995a, b). Notably, cantharidin did not increase the beating rate in right atrial preparations. This can be observed in the original recording presented in Fig. 4A. It can be argued that our system is not sufficiently sensitive for detecting changes in the beating rate. However, this explanation of our findings is unlikely because the additional isoprenaline applied to the samples elevated the spontaneous beating rate in these right atrial preparations. Several such experiments are summarized in Fig. 4B. Moreover, we studied the effects of cantharidin on the positive inotropic effects of isoprenaline. It turned out that per-incubation of the mouse left atrial preparations with 30 µM cantharidin shifted the concentration response curve of isoprenaline to the left name from (- lg EC_50_-values) 7.44 ± 0.07 to 8.53 ± 0.46 (*n* = 3–4, *p* < 0.05).

**Fig. 5:**
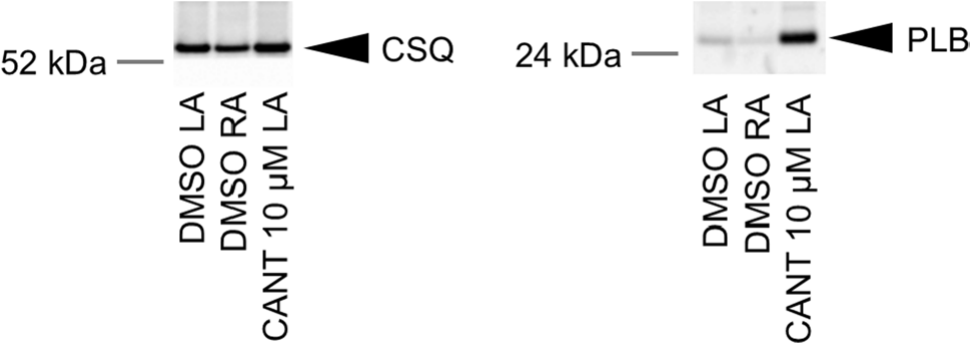
Cantharidin increases phosphorylation in mouse atrium. Cantharidin increases phosphorylation in isolated mouse atrium. Contracting preparations as seen in Fig. 2 were freeze clamped. Typical Western blots are seen Western blots depict phospholamban with arrows. As a loading control, we assessed the protein expression of calsequestrin (CSQ) by cutting the lanes of the blot and incubating the lower and upper halves with different primary antibodies. Concentration of cantharidin (CANT) is indicated. Molecular weight markers are indicated with arrows and marked with kilo Dalton (kDa)

As a next step towards a deeper understanding of the biochemical mechanism underpinning these mechanical responses in mouse atrium, we investigated whether cantharidin increased cardiac phosphorylation in a mouse atrium. Therefore, at the end of the contraction experiments illustrated in Fig. 2A, we froze the mouse atrial samples and performed Western blotting. It is apparent that 10 µM cantharidin increased the phosphorylation state of phospholamban in the mouse atrium (Fig. 5), which is similar to our previous findings on guinea pig ventricular cardiomyocytes (Neumann et al. 1995a, b). In more detail, we quantified the phosphorylation state of the proteins of interest in arbitrary numbers and divided each value by the expression of calsequestrin the same protein lane. Using this normalization, we noted that 100 µM cantharidin increased the phosphorylation state of PLB at amino acid 16 from 0.57 ± 0.17 to 4.27 ± 0.42 (*n* = 3, each, *p* < 0.05) and the phosphorylation state of myosin light chain 20 from 0.87 ± 0.22 to 5.40 ± 1.13 (*n* = 3, each, *p* < 0.05).

Having studied the mechanical and biochemical effects of cantharidin in mouse atrial preparations in some detail, it seems more clinically relevant to consider similar studies on atrial preparations from humans. As we had already published a study on the contractile effects of cantharidin in failing and non-failing human ventricular muscle strips (Linck et al. 1996), a major remaining open question was how cantharidin might act in human atrial preparations. Specifically, would cantharidin be as potent in the human ventricle as in the mouse atrium or much less potent? In essence, we would be looking for species- or region-specific differences in the action of cantharidin in mice and humans.

**Fig. 6:**
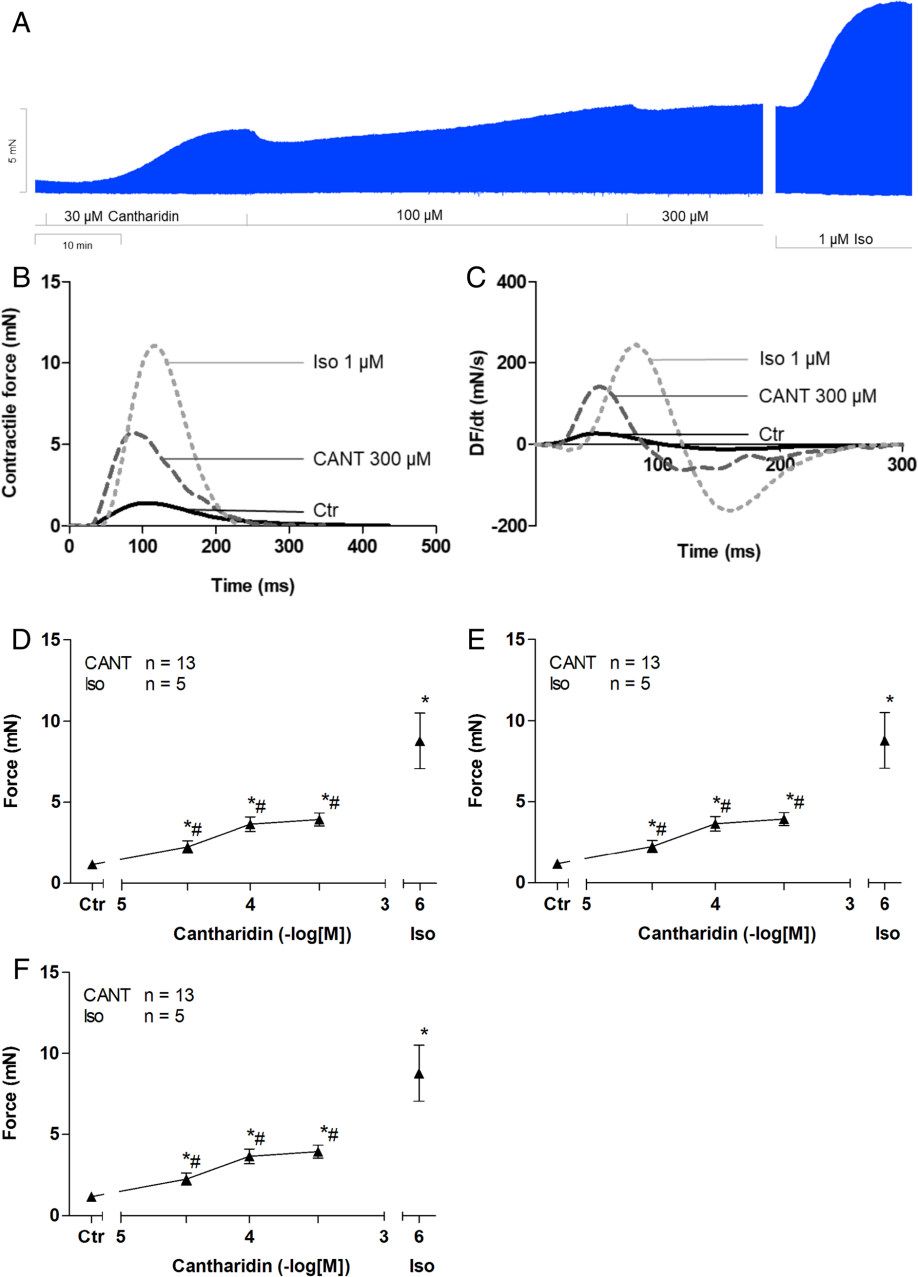
Cantharidin increases force in the human atrium**.** A: Original recording of the concentration- and time-dependent positive inotropic effect of cantharidin in milli Newton (mN, Ordinate) (min, horizontal bar). In some experiments, samples were fast frozen in liquid nitrogen for measurement of protein phosphorylation. Note that the increase in force is slow compared to isoprenaline. In some samples we noted an increase in diastolic tension typical of contractures. In some samples we added at the end of the experiment 1 µM isoprenaline to the organ bath to test for efficiencies. B: Original recordings superimposed at high temporal resolution in milli seconds (ms). Here, the increase in force by cantharidin compared to unstimlated contraction (CTR). Please note that cantharidin while increasing force also shortens relaxation compared to CTR. For comparison the effect of isoprenaline is shown. C: Force of contraction, D: time to peak tension (T1), D: time to relaxation (T2), E: rate of contraction (dF/dt_max_), E: rate of relaxation (dF/dt_min_). * *p* < 0.05 vs. CTR, # *p* < 0.05 vs. isoprenaline (Iso). Ordinate in 6A and 6B: Force of contraction in milli Newton (mN). Ordinate in 6 A, 6B, 6C and 6D in mN. Ordinate in 6E in milli seconds (ms). Rate of contraction and rate of relaxation in 6F mN/ms. Ascissae indicate concentrations of cantharidin in negative decadic concentrations. Horizontal bar in A indicates time axis in minutes (min). Signficant difference versus control (CTR; pre-drug value) is indicated in asterisks. “n” indicates number of experiments

Similar to the findings in mouse atria, we also observed that cantharidin increased the force of contraction in a concentration- and time-dependent manner in the human atrium, as seen in the original recording presented in Fig. 6A. The inotropic effect of cantharidin became observable at higher concentrations in the human atrium than was observed in the mouse atrium (Fig. 2D), but at similar concentrations observed in the human ventricle (Linck et al. 1996). The data on these positive inotropic effects are plotted as single fast single contractions in the human atrium (Fig. 6B). The increase in the force of contraction is accompanied by an increase in the rate of relaxation and in the rate of tension development, as seen in the original recording presented in Fig. 6C and is also discernible in the summarized data presented in Fig. 6F. The positive inotropic effect of cantharidin became significant at concentrations of 30 µM and higher in right atrial preparations from humans (Fig. 6D). Cantharidin (at 300 µM) was less effective than 1 µM isoprenaline at raising the force of contraction in right atrial preparations from humans (Fig. 6A, 6D). Furthermore, although cantharidin failed to shorten the time to peak tension, it shortened the time to relaxation in the human atrium (Fig. 6), which is similar to our observations on the human ventricle in a previous study (Link et al. 1996). Moreover, we studied the effects of cantharidin on the positive inotropic effects of isoprenaline. It turned out that per-incubation of the human right atrial preparations with 100 µM cantharidin shifted the concentration response curve of isoprenaline to the left namely from (- lg EC 50 values) 8.42 ± 0.16 to 9.35 ± 0.20 (*n* = 3–4, *p* < 0.05).

**Fig. 7:**
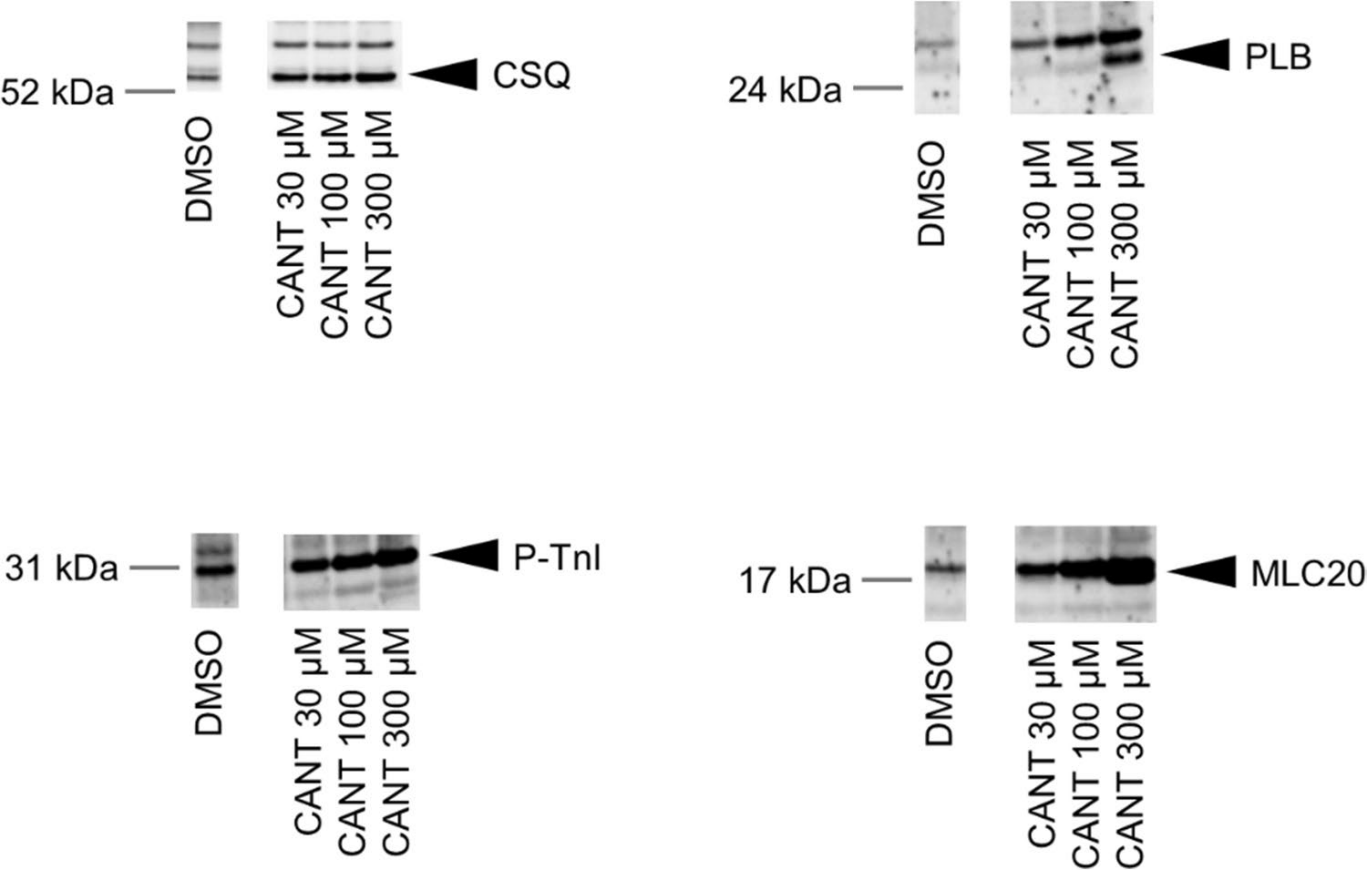
Cantharidin increased phosphorylation. Cantharidin increases phosphorylation in isolated human atrium. Contracting preparations as seen in Fig. 6A were freeze clamped. Typical Western blots are seen Western blots depict phosphorylated phospholamban, phosphorylated TnI and phosphorylated MLC20 with arrows. As a loading control, we assessed the protein expression of calsequestrin (CSQ) by cutting the lanes of the blot and incubating the lower and upper halves with different primary antibodies. Concentration of cantharidin (CANT) is indicated in micro molar concentration (µM). Molecular weight markers are indicated with arrows and marked

It is apparent that 100 µM cantharidin increases the phosphorylation state of phospholamban (PLB), the inhibitory subunit of troponin (Tnl) and myosin light chain 20 (MLC20) in the human atrium (Fig. 7), which is in agreement with our previous study on guinea pig ventricular cardiomyocytes using a different methodology (Neumann et al. 1995a, b). In more detail, we quantified the phosphorylation state of the proteins of interest in arbitrary numbers and divided each value by the expression of calsequestrin the same protein lane. Using this normalization, we noted that 300 µM cantharidin increased the phosphorylation state of PLB at amino acid serine 16 from 0.42 ± 0.09 to 2.68 ± 0.60 (*n* = 4, each, *p* < 0.05), the phosphorylation state of myosin light chain 20 from 0.88 ± 0.16 to 5.25 ± 0.83 (*n* = 4, each, *p* < 0.05) and the phosphorylation state of TnI from 0.51 ± 0.14 to 1.24 ± 0.11 (*n* = 4, each, *p* < 0.05).

## Discussion

### Main new findings

The primary new findings in this study comprise a positive inotropic effect of cantharidin accompanied by an increase in protein phosphorylation in the human atrium. Similarly, a positive inotropic effect of cantharidin in mouse atria and an increase in protein phosphorylation in the presence of cantharidin in mouse atria have not been previously reported.

### Cantharidin mechanism of action

Our assumption is that, similar to its effect in the human ventricle, cantharidin also inhibits PP1 and/or PP2A activity in the human atrium. We have shown previously, on mRNA and at the level of proteins, that the catalytic subunits of PP1 and PP2A are present in the human atrium (Lüss et al. 2000); hence, cantharidin targets are present in the human atrium. The potency of cantharidin in inhibiting PP1 and PP2A in the human heart in vitro is so similar that the experiments in this study do not allow us to discriminate between the functional roles of PP2A and PP1 in human atrial preparations with regard to an increase in the force of contraction (Neumann et al. 1995a, b; Knapp et al. 1998a, b; Neumann et al. 1999). Numerically, cantharidin is slightly more potent at inhibiting PP2A than PP1 (Neumann et al. 1995a, b); however, this does not support the interpretation that the positive inotropic effect of cantharidin at low concentrations is due to the inhibition of PP2A, while at high cantharidin concentrations, it is due to the inhibition of PP1. What seems apparent is that PP1 and PP2A each account for about 45% of PP activity in the human heart; combined, they account for approximately 90% of PP activity in the human heart (Herzig and Neumann 2000). Thus, it seems reasonable to conclude that cantharidin acts by inhibiting PP1 and PP2A in concert. Thereby, cantharidin increases the phosphorylation of phospholamban, TnI and MLC20 at the very least. We have previously shown that incubation with cantharidin increases the phosphorylation of many proteins in radioactively labelled guinea pig ventricular cardiomyocytes (Neumann et al. 1995a, b). It is also clear that human phospholamban is dephosphorylated by PP1, as well as by PP2A (Neumann et al. 1999). Previously, we performed autoradiography on dried radioactive gels from ^32^P-labelled guinea pig cardiomyocytes to detect cantharidin-induced protein phosphorylation (Neumann et al. 1995a, b). Our current protocol for multicellular human (or mouse) atrial preparations does not use radioactively labelled proteins; it uses phosphorylation-specific antibodies. This approach has the advantage of ensuring that no radioactivity is used in the laboratory. However, the disadvantage is that we cannot trace the complete pattern of protein phosphorylation in the human atrium.

### Role of phosphorylation of regulatory proteins

The general assumption is that the phosphorylation of phospholamban can explain the reduced time to relaxation and the increased rate of tension relaxation in cardiac muscles following β-adrenergic stimulation of the mammalian heart. This occurs because Ca^2+^ is speedily transported via sarco/endoplasmic reticulum Ca^2+^-ATPase (SERCA) back into the sarcoplasmic reticulum (SR), and because there is now more Ca^2+^ in the SR, the next heartbeat can release more Ca^2+^ from the SR (Fig. 1). Hence, phospholamban phosphorylation is thought to also contribute to the positive inotropic effect of cantharidin and an increase in the rate of tension development in the presence of cantharidin (Boknik et al. 2001). In patch-clamp experiments, cantharidin in isolated guinea pig ventricular cardiomyocytes increases the current flowing through the L-type Ca^2+^ channel, and this increase in the current is effected via cAMP-dependent phosphorylation (Neumann et al. 1995a, b, Boknik et al. 2000). It is generally accepted that not PP1 and/or PP2A, but also calcineurin (PP2B), are involved in the role of phosphatases in dephosphorylation in the L-type Ca^2+^ channel in human atrial cardiomyocytes. Hence, we assume that cantharidin would also increase this current in mouse and human atria; however, this remains to be confirmed via further experimentation. Notably, the increase in MLC20 phosphorylation needs to be addressed. The increase in the phosphorylation state of MLC20 by inhibitors of phosphatases is not unexpected. Using radioactive methods in previous research, we detected an increase in the phosphorylation state of MLC20 in guinea pig ventricular cardiomyocytes in the presence of cantharidin (Knapp et al. 1998a, b) and in the presence of two other well-studied phosphatase inhibitors: calyculin A (Neumann et al. 1994; Neumann et al. 1995a, b) and okadaic acid (Neumann et al. 1993). Calyculin A and okadaic acid were not used in this study because of their prohibitive costs when procured for organ bath experiments (volume 10 ml).

### The special role of MLC20 phosphorylation

MLC20 is not a substrate of PKA in vitro or in vivo. In vivo, stimulation of PKA via isoprenaline application leads to an increase in the phosphorylation states of phospholamban and TnI in perfused guinea pig hearts and guinea pig ventricular cardiomyocytes. MLC20 has been studied extensively in smooth muscle cells. A study has clearly established that MLC20 is usually phosphorylated by an MLC kinase and not by PKA; there are data showing that MLC20 is dephosphorylated by both PP1 and PP2A (Chang et al. 2016; Sheikh et al. 2015; Kitazawa et al. 2021).

Based on our previous data on radioactively labelled cardiomyocytes (Neumann et al. 1995a, b), we predict that, using other phosphorylation-specific antibodies for other antigens or using phosphorylation-specific mass spectroscopy methods, many more phosphorylated proteins would be detectable in human atria incubated with cantharidin.

### Species differences

Notably, cantharidin is more potent at increasing the force of contraction in mouse atria than in human atria. This finding may also support the assumption that cantharidin increases contractile force via phosphatase inhibition. In guinea pig cardiac preparations (Neumann et al. 1995a, b), as in mouse cardiac preparations, cantharidin was more potent at increasing contractile force than in human ventricular preparations; in line with these mechanistic observations, cantharidin is more potent at inhibiting phosphatase activity in homogenates from guinea pig ventricles than homogenates from human ventricles (Neumann et al. 1995a, b).

### Effects on beating rate

Regarding our findings on mouse right atrial preparations, some authors have presented good data, albeit on rabbit sinus node cells, that phosphorylation—of phospholamban especially—is necessary for an increase in beating rate after β-adrenergic stimulation of sinus node cells (review: Vinogradova and Lakatta 2021). Our data on mice (presented in this study) and on guinea pigs (Neumann et al. 1995a, b) are in disagreement with theirs, as we did not detect an increase in beating rate with cantharidin, which may very well be due to species differences. Furthermore, it would be crucial to support or refute our data on cantharidin in human sinus node cells; however, this is beyond the scope of this study. In addition, together with other researchers, we have generated mice with overexpressed, knocked out, or knocked down cardiac phosphatases. Based on our initial data on mice presented in this study, it may be useful to test our assumptions by studying cantharidin in these generated transgenic mice. We are currently planning such studies on our transgenic models.

### Clinical relevance

Our current data confirm and extend our previous data that phosphatase inhibition may be a potential treatment modality for heart failure. However, as we have shown in previous studies, cantharidin induces vasoconstriction in isolated animal coronary muscle strips (Knapp et al. 1997; Knapp et al. 1998a, b) and human coronary muscle strips (Knapp et al. 1999). Hence, cantharidin in humans would increase the force of contraction in the cardiac muscles; however, cantharidin would simultaneously reduce or even stop coronary perfusion. Hence, it now remains for organic chemists to synthesize cardiomyocyte-specific phosphatase inhibitors that would increase the force of contraction without reducing coronary flow or harming other organs. Another caveat for further research building on this study lies in the species differences between mice and humans with regard to cantharidin. Our submitted observation remains that cantharidin is much more potent at increasing the force of contraction in mouse cardiac preparations than in human cardiac preparations. This shows that new PP inhibitors must be tested in human tissue, conceivably in stem cells presently derived from human cardiomyocytes.

### Limitations of the study

We did not measure phosphorylation in the human left ventricle. The human left ventricle is more important for force generation than the human right atrium. Regrettably, human ventricular samples are currently unavailable to us. Furthermore, it can be argued that we did not measure phosphorylation in human cardiomyocytes. It is conceivable that cantharidin inhibits PP not only in cardiomyocytes, but also in other cells in the human heart, such as endothelial cells or smooth muscle cells. This is certainly a possibility for MLC20 because MLC20 occurs not only in cardiomyocytes but also in other cell types. However, we argue that phospholamban and TnI are cardiomyocyte-specific proteins; hence, an increase in their phosphorylation is, in all likelihood, due to the inhibition of PP in cardiomyocytes by cantharidin. Upon carefully observing the original recordings of human cardiomyocytes, a transient fall in the force of contraction can be observed at cantharidin concentrations of 100 µM and 300 µM. We surmise that this transient drop in the force of contraction is attributable to the solvent used. We used a 100 mM stock solution of cantharidin in DMSO. Thus, the human samples required more cantharidin than the mouse atria for an increase in contractile force. Therefore, the human atria also received more DMSO than the mouse atria. We observed this decline in contractile force induced by cantharidin in a previous study on human cardiac tissue (the ventricle) with cantharidin dissolved in DMSO (Linck et al. 1996).

In summary, we have validated the hypotheses put forward in the Introduction as follows: (1) Cantharidin increases the phosphorylation of vital regulatory proteins in the human atrium. (2) Cantharidin increases the force of contraction in the human atrium. (3) Cantharidin increases relaxation in the human atrium, which is probably attributable to enhanced phosphorylation of phospholamban and Tnl.

## Data Availability

The data of this study are available from the corresponding author upon reasonable request.
